# *Dichrocephala integrifolia* Aqueous Extract Antagonises Chronic and Binges Ethanol Feeding-Induced Memory Dysfunctions: Insights into Antioxidant and Anti-Inflammatory Mechanisms

**DOI:** 10.1155/2022/1620816

**Published:** 2022-09-06

**Authors:** Bertrand Yuwong Wanyu, Nadège Emégam Kouémou, Germain Sotoing Taiwe, Gwladys Temkou Ngoupaye, Linda Tamanji Ndzweng, Agathe Lambou Fotio, Mireille Sylviane Nguepi Dongmo, Elisabeth Ngo Bum

**Affiliations:** ^1^Department of Animal Biology and Conservation, Faculty of Science, University of Buea, P.O. Box 63, Buea, Cameroon; ^2^Department of Animal Biology, Faculty of Science, University of Dschang, P.O. Box 67, Dschang, Cameroon; ^3^Department of Biochemistry and Molecular Biology, Faculty of Science, University of Buea, P.O. Box 63, Buea, Cameroon; ^4^Department of Biological Sciences, Faculty of Science, University of Maroua, P.O. Box 52, Maroua, Cameroon

## Abstract

Ethanol consumption is widely accepted despite its addictive properties and its mind-altering effects. This study aimed to assess the effects of *Dichrocephala integrifolia* against, memory impairment, on a mouse model of chronic and binges ethanol feeding. Mice were divided, into groups of 8 animals each, and received distilled water, *Dichrocephala integrifolia* aqueous extract (25; 50; 100; or 200 mg/kg) or memantine (200 mg/kg) once a day, while fe, with Lieber-DeCarli control (sham group only) or Lieber-DeCarli ethanol diet ad libitum for 28 days. The *Y* maze and the novel object recognition (NOR) tests were used to evaluate spatial short-term and recognition memory, respectively. Malondialdehyde, nitric oxide, glutathione levels, and proinflammatory cytokines (Il-1*β*, TNF-*α,* and Il-6) were evaluated in brain homogenates following behavioral assessments. The results showed that chronic ethanol administration in mice was associated with a significant (*p* < 0.001) reduction in the spontaneous alternation percentage and the discrimination index, in the *Y* maze and the NOR tests, respectively. It significantly (*p* < 0.01) increased oxidative stress and inflammation markers levels in the brain. *Dichrocephala integrifolia* (100 and 200 mg/kg) as well as memantine (200 mg/kg) significantly (*p* < 0.001) increased the percentage of spontaneous alternation and the discrimination index, in the *Y* maze and NOR tests, respectively. *Dichrocephala integrifolia* (100 and 200 mg/kg) likewise memantine (200 mg/kg) significantly (*p* < 0.01) alleviated ethanol-induced increase, in the brain malondialdehyde level, nitric oxide, Il-1*β*, TNF-*α,* and Il-6. From these findings, it can be concluded that *Dichrocephala integrifolia* counteracted memory impairment, oxidative stress, and neuroinflammation induced by chronic ethanol consumption in mice.

## 1. Introduction

Ethanol is a psychoactive substance of common abuse widely accepted in different cultures [1–3]. Despite its addictive properties and its mind altering-effects, ethanol is a drug with little or no stigma [4–6]. The global incidence of alcoholism is increasing and Africa bears the heaviest burden of neurological disorders attributed to alcohol, mainly because, many African countries lack efficient public policies that regulate the trading and consumption of ethanol [1]. Chronic ethanol consumption is the leading cause of depression, anxiety, addiction, mental retardation, and learning and memory dysfunctions [2, 7–11], Long term misuse of ethanol can lead to permanent hippocampal and/or cortex damages [12] that correlates with corresponding memory disorders [13, 14]. Chronic ethanol exposure has been proven to impair hippocampus spatial learning and memory in rodents as well as in humans [13, 15–19]. Ethanol acts on numerous cellular and molecular targets within the brain. The real mechanism by that ethanol leads to memory dysfunctions is poorly understood [20]. Nevertheless, myriad studies have tried to explain the mechanisms by which alcohol induces neurotoxicity and also give a direction of treatment [21, 22]. Ethanol causes an over-excitation of N-methyl-D-aspartate receptors of glutamate leading to subsequent excitotoxicity, oxidative stress, neuroinflammation, and neurodegeneration [9, 22, 23].

There are different attempts to solve disorders resulting from alcoholism. Treatment options include psycho-social interventions and the use of medications [24]. These medications either prevent withdrawal symptoms or reverse the cognitive deficits resulting from ethanol misuse [21, 23]. Drugs treatment strategies include benzodiazepines (disulfiram, piracetam, diazepam, etc.) or N-methyl-D-aspartate antagonist (memantine). These medications in addition to the fact that there are expensive, and associated with various side effects, do not target all features of human alcoholism [24, 25]. Therefore, there is an urgent need to develop new, affordable, and safer treatments against ethanol-induced cognitive deficits. Different molecules from plants and plant extracts have been acknowledged for their potential to mitigate ethanol toxicity due to either their anti-oxidant and/or their anti-inflammatory properties [9, 23].

*Dichrocephala integrifolia* is a plant belonging to the family Asteraceae, and that is widely distributed in Africa, Asia, Turkey-Europe, and the Middle East [26]. The various uses of this plant in traditional medicine have been commonly reported in many countries. In Kenya, *Dichrocephala integrifolia* is called “ekengeta mbori” and its leaf infusion is used by traditional healers in the treatment of oxidative stress disorders and memory loss [27]. It is also reported that in Ethiopia, *Dichrocephala integrifolia* is a traditional remedy for the treatment of wound infections and other ailments [28]. This plant is known as “vawk-ek-a-tum-tual” in India (mizo) and it is decoction is used to treat kidney diseases, cancer, and microbial infections [29]. The population of Nepal and Yemen uses *Dichrocephala integrifolia* in the treatment of cancer and infectious diseases [30, 31]. In Cameroon, this plant locally known as “mbag,” “api,” “ngninada elokn,” “kévé,” and “ganki” is used as a therapy for fever, diarrhea, hypertension, malaria, asthma, inflammatory diseases, stomach ulcers, memory impairment and liver dysfunctions [32–36]. The chemical constituents of *D. integrifolia* includes: diterpene lactones, sesquiterpenes lactones, sterols, dichrocephol, dicaffeoylquinic, saponins, alkaloids anthraquinones, and flavonoids [26, 37]. A study conducted by Ngueguim and collaborators in 2016, showed that *D. integrifolia* leaves' aqueous extract prevents liver damage induced by chronic ethanol administration in rats [33]. Therefore, we hypothesized that an aqueous extract prepared from the leaves of *D. integrifolia* can be a good pharmacotherapeutic candidate to mitigate alcohol-induced neurotoxicity. Thus, the aim of the present study was to evaluate the effects of the aqueous extract prepared from the leaves of *D. integrifolia* on chronic ethanol feeding and ethanol binges-induced memory impairment, oxidative stress, and neuroinflammation in mice.

## 2. Materials and Methods

### 2.1. Plant Material and Extract Preparation

The leaves of *D. integrifolia* used in this study were harvested in Buea (Cameroon) in February 2021. The confirmation of the Species was done at the National Herbarium of Cameroon with voucher specimen number: HNC-Num.50074/HNC. The preparation of the plant extract was done as previously described by Kouémou et al. with little modifications [32]. Briefly, the leaves were washed and shade-dried for two weeks at room temperature. The dried leaves were then ground in a blender. One hundred grams (100 g) of the dried fine powder was infused into 750 mL of distilled water. The mixture was boiled for 20 minutes, allowed to cool, and then filtered using Whatman No1 filter paper. The filtrate was oven-dried at 40 C to obtain a brown extract of 6.25 g, the yield of extraction was thus 6.25%. From it, the stock solution was prepared and later on diluted to give the four doses of the plant extract (25 mg/kg, 50 mg/kg, 100 mg/kg, and 200 mg/kg) used. The doses of the plant were selected according to our previous studies and to the literature [32, 33].

### 2.2. Animals and Ethical Consideration

A total of 56 young adult Swiss mice; males (42) and females (14) aged two months and weighing between 20 and 24 g were used. These animals were raised in the Animal House of the Faculty of Science of the University of Buea (Cameroon) where they were kept in standard cages at room temperature. All mice were acclimatized to laboratory conditions for one week prior to the start of the experiments. All experiments were conducted in accordance with the guide for the Care and Use of Laboratory Animals published by the National Institute of Health (NIH publication No. 85–23, revised1996). This study was approved by the University of Buea-Institutional Animal Care and Use Committee (UB-IACUC) with the permit number N: UB-IACUC N 8/2021.

### 2.3. Drugs and Chemicals

Memantine was purchased from MedChemicExpress; whereas, ethanol 95% was procured from a local pharmacy. Also, trichloroacetic acid, thiobarbituric acid, sulphanilamide, naphthalenediimide, phosphoric acid, and Ellman reagent were all purchased from Sigma-Aldrich (Germany); while Lieber-DeCarli ‘82 ethanol (Product F1258SP) and Lieber-DeCarli ‘82 control (Product F1259SP) diet, as well as Maltose dextrin (Product 3585), were from Bio-Serv (United States). ELISA Kits for the quantification of tumor necrosis factor-*α* (TNF-*α*), interleukin-1*β* (IL-1*β*), and interleukin-6 (IL-6) were from R&D systems (United States).

### 2.4. Animal Grouping and Chronic Ethanol Feeding

Mice were randomly divided into seven (7) groups of eight (8) mice (6 males and 2 females) each as shown in Table 1.

**Figure 1:**
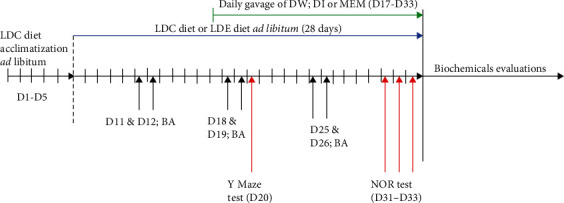
Overview of the experimental procedure. BA: binge administration, D1 to D33: Corresponding days of the experiment; DI: *Dichrocephala integrifolia*; DW: distilled water; LDC: Lieber-DeCarli control; LDE: Lieber-DeCarli ethanol; MEM: Memantine; NOR: Novel object recognition.

The mouse model of chronic ethanol feeding plus binges (the National Institute on Alcohol Abuse and Alcoholism (NIAAA) model) with minor modifications was used in this study. After grouping, all mice were fed with Lieber-DeCarli control (LDC) diet ad libitum for five days to acclimatize animals to the feeding tubes. After the acclimatization phase, mice in the sham group received LDC diet for the rest of the experimentation phase, while mice in the other groups received Lieber-DeCarli ethanol (LDE) diet containing 5% alcohol and maltose dextrin. In addition, they also received ethanol (20% (v/v)) binges through gavage twice a week. The animals in the sham group received a comparable volume of distilled water (10 mL/kg) through gavage. The total duration of administration was 33 days (Figure 1).

### 2.5. BehavioralAssessments

The *Y* maze was used to evaluate spatial short-term memory on day 20 and the novel object recognition (NOR) test to evaluate recognition memory between days 31 and 33 (Figure 1).

#### 2.5.1. The Y-Maze Test

The *Y*-maze test was used to assess spatial short-term memory of mice on a single-day test session as earlier described by Kraeuter and coworkers in 2019 [38]. The maze used in this work was a locally fabricated Plyboard maze. The *Y*-maze consisted of a *Y*-shaped apparatus of three analogous branches (35 cm length, 8 cm height, and 15 cm width) attached in a single piece. The angle between two branches of the maze was 120°. Three letters A, B, and C were used to differentiate each arm of the maze [32, 38, 39]. One hour after treatment on day 20, each naive mouse to the maze was placed in one of the three starting arms for an exploration session of 8 minutes. The number of arms entries, as well as spontaneous alternations, were recorded by an experienced researcher blind to the treatments. Spontaneous alternation refers to a consecutive entry into the three different branches of the maze in any order (ABC, BCA, or CAB) [32, 38, 39]. Before the passage of the next mouse, the maze was thoroughly cleaned with 5% ethanol to remove residual smells.

The percentage of spontaneous alternation is defined as: (number of alternations)/(total arms entries-2) *X* 100.

#### 2.5.2. Novel Object Recognition Test

The NOR test was used, to evaluate recognition memory in mice between days 31 to 33. This test was performed in an open field. The open field used had the following dimension 40 cm length *x* 40 cm width *x* 25 cm high. On day 31 during the habituation phase of the test, each mouse individually was familiarized with the maze for 5 minutes, to avoid neophobia-induced stress. The acquisition and retention phases were performed 1 hour after treatment on days 32 and 33, respectively. During the retention phase, two identical objects (*A* + *A*) were presented to each animal for a 5 minutes exploration session. Exploration is considered when an animal touches the object or points its nose less than 2 cm towards the object. The following day, one of the objects presented during the acquisition phase was replaced by a new object (B). In this phase as in the previous one, each single animal was allowed to explore the two objects (*A* + *B*) for 5 minutes. The time taken by the animal to explore object A or object B was recorded as TA and TB, respectively. A discrimination index (DI) was calculated for each animal using the formula (DI = (TB − TA)/(TB + TA) [40–42].

### 2.6. Biochemical Assessments

Following the retention phase of the NOR, all the mice were fasted overnight and humanely sacrificed by decapitation after light ether anesthesia. The brains were quickly removed from the skulls and directly used for the preparation of homogenates.

#### 2.6.1. Preparation of Brain Homogenates

Each whole brain was weighed and rinsed with NaCl 0.9%, then crushed into a mortar to prepare 20% homogenate (w/v) using 50 mm Tris buffer. The homogenates were then centrifuged at 3000 rpm for 20 min. The supernatants collected were stored at −20°C and later used for the evaluation of oxidative stress parameters and proinflammatory cytokines.

#### 2.6.2. Determination of Pro Inflammatory Cytokines

Brain levels of TNF-*α*, IL-1*β,* and IL-6 were evaluated using mouse ELISA kits (Quantikine ELISA kits; R&D Systems Biotech, USA) according to the manufacturer's instructions. Succinctly, 50 *μ*L of brain homogenates or standards were pipetted into precoated 96-wells microplates. The antibodies cocktails from the kits were then added to the wells. After incubation, at room temperature for two hours, the microplates were washed with the wash buffer. Subsequently, the substrate solution was added to each well followed by incubation for 30 min in the dark at room temperature. The stop solution was added to stop the reaction. The optical density was read at 450 nm using a microplate reader (Thermo Scientific; MultiskanTM FC Microplate Photometer). The concentration of cytokines is expressed in pg/mL.

#### 2.6.3. Determination of Brain Nitric Oxide Level

Brain nitric oxide level was measured in the brain using the Greiss reagent [43, 44]. Briefly, 0.5 mL of Greiss reagent and 0.5 mL of brain's supernatant were introduced into a test tube and allowed to stand for 5 minutes, after which the absorbance of the mixture was read at 540 nm using a spectrophotometer (Thermo Scientific; MultiskanTM FC Microplate Photometer). Nitric concentration expressed in mol/g of tissue sample was determined using sodium nitrate standard curve.

#### 2.6.4. Assessment of Brain Malondialhydehyde (MDA) Level

Brain's lipid peroxidation level was measured through the thiobarbituric assay [45]. A volume of 0.25 mL of trichloroacetic acid (20%) and 0.5 mL of thiobarbituric acid (0.67%) added to 0.5 mL of the brain sample. The mixture was incubated for an hour in a water bath at 90°C. After cooling with tap water, the samples were centrifuged for 10 minutes at 3000 rpm. The optical density of the supernatant was read using a spectrophotometer (Thermo Scientific; MultiskanTM FC Microplate Photometer) at 546 nm. The brain level of MDA expressed in *μ*mol/g of tissue was calculated using Beer Lambert's formula and the extinction coefficient of:1.6 × 105 M/cm [46].

#### 2.6.5. Evaluation of Reduced Glutathione Level

The brain level of reduced glutathione (GSH) was determined using Ellman's reagent as originally described by Ellman [47] with little modifications. A volume of 1.5 mL of Ellman's reagent was added to 100 *μ*L of brain sample and kept at room temperature for 60 minutes, after which the absorbance was read at 405 nm using a spectrophotometer (Thermo Scientific; MultiskanTM FC Microplate Photometer) [48].

#### 2.6.6. Assessment of Catalase Activity

The protocol described by Fotio et al. was used to evaluate catalase activity in the brain samples. Briefly, 125 *μ*L of supernatant was added to 125 *μ*L of 0.1 M phosphate buffer (pH 7.4) and 0.5 mL of 30 mM of hydrogen peroxide (H2O2). The absorbance was read at 240 nm for 30 s, 60 s, and 90 s using a spectrophotometer (Thermo Scientific; MultiskanTM FC Microplate Photometer). Catalase activity was expressed as mmol of g of tissue [46].

### 2.7. Statistical Analysis

Data collected were entered into spreadsheets using Microsoft Excel and expressed as mean ± standard error of the mean (SEM) or as a percentage. Statistical differences between groups were calculated by one-way analysis of variance (ANOVA), followed by Turkey's multiple comparison test. The statistical package used was graph pad prism 8.4.3(686). The differences were considered significant at *p* < 05.

## 3. Results

### 3.1. Effects of *D. Integrifolia* on Spontaneous Alternation and Locomotion of Alcohol-Treated Mice in the Y Maze

**Figure 2:**
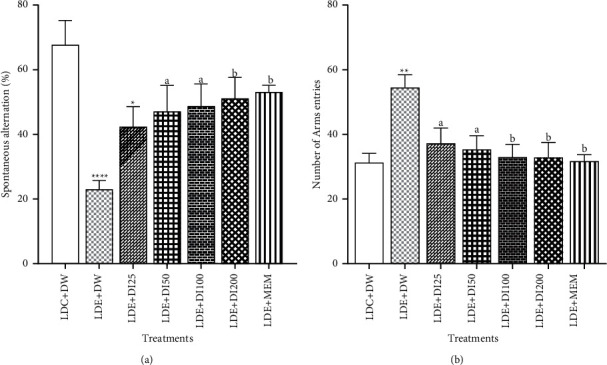
Effects of *D.* integrifolia on spontaneous alternation percentage (a) and the number of arms entries (b) of alcohol-treated mice. Each bar represents the mean ± S.E.M, *n* = 8. Data were analyzed with the ordinary One-Way ANOVA followed by Tukey multiple comparisons test.^*∗*^*p* < 0.05, ^*∗∗∗*^*p* < 0.01, ^*∗∗∗*^*p* < 0.001, vs. LDC + DW group and ^a^*p* < 0.05, ^b^*p* < 0.01, vs LDE + DW group. DI25 : *Dichrocephala integrifolia* 25 mg/kg, DI50 : *Dichrocephala integrifolia* 50 mg/kg, DI100 : *Dichrocephala integrifolia* 100 mg/kg, DI200 : *Dichrocephala integrifolia* 200 mg/kg, DW: distilled water (10 mL/kg), LDC : Lieber-DeCarli control diet, LDE : Lieber-DeCarli ethanol diet, MEM : Memantine (20 mg/kg).

Chronic ethanol feeding and ethanol binges significantly (*p* < 0.001) reduced the percentage of spontaneous alternations in the sham (LDE + DW) group compared to the negative (LDC + DW) control group (Figure 2(a)). The percentage of spontaneous alternation decreased from 67.77 ± 7.48% in the LDC + DW group to 23 ± 2.7% in the LDE + DW group. *D. integrifolia* (100 and 200 mg/kg) as well as memantine significantly (*p* < 0.05) increased the spontaneous alternation percentage (Figure 2(a)). As shown in Figure 2(b), *D. integrifolia* and memantine, significantly decreased (*p* < 0.05) the number of arm entries in the *Y* maze when compared to the LDE + DW-treated group. The number of arms entries decreased (*p* < 0.01) from 54.5 ± 3.97 in the LDE + DW-treated group to 32.88 ± 4.63 and 31.75 ± 1.98 in the mice treated with the plant extract (200 mg/kg) and memantine (20 mg/kg), respectively (Figure 2(b)).

### 3.2. Effects of *D. Integrifolia* on the Exploration times and the Discrimination Index of Alcohol-Treated Mice in the Novel Object Recognition Test

**Figure 3:**
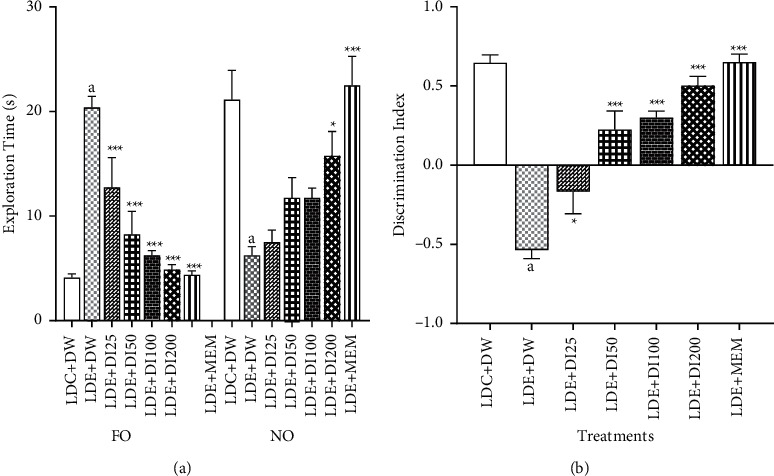
Effects of *D. integrifolia* on the discrimination index of alcohol-treated mice. Each bar represents the mean ± S.E.M, *n* = 8. Data were analyzed with the ordinary one-way ANOVA followed by Tukey multiple comparisons test. ^*∗∗∗*^*p* < 0.0001, ^*∗*^*p* < 0.05 vs LDE + DW group ^a^*p* < 0.0001 vs LDC + DW group. DI25: *Dichrocephala integrifolia* 25 mg/kg, DI50: *Dichrocephala integrifolia* 50 mg/kg, DI100: *Dichrocephala integrifolia* 100 mg/kg, DI200: *Dichrocephala integrifolia* 200 mg/kg, DW: distilled water (10 ml/kg), FO: Familiar object, LDC: Lieber-DeCarli control diet, LDE: Lieber-DeCarli ethanol diet, MEM: Memantine (20 mg/kg), NO: Novel object.

Figure 3(a) depicts the exploration time of the novel object (NO) and the familiar (FO) object during the retention phase of the NOR test. Ethanol administration increased the exploration of the familiar object and decreased the exploration of the novel object when compared to the sham group. *D. integrifolia* (100 mg/kg and 200 mg/kg) likewise memantine, significantly (*p* < 0.01) increased the exploration of the novel object and reduced that of the familiar object. The exploration time of the novel object raised from 6.25 ± 0.84 in the negative control to 11.8 ± 0.92; 15.8 ± 2.33 and 22.5 ± 2.76 in the groups treated with the plant extract at doses of 100 and 200 mg/kg and memantine, respectively (Figure 3(a)).

As presented in Figure 3(b), chronic ethanol feeding with ethanol binges lead to a decrease in the recognition index of the animals in the LDE + DW-treated group. The discrimination index dropped from 0.64 ± 0.05 in the LDC + DW-treated group to −0.53 ± 0.05 in the LDE + DW-treated group. Treatment of mice with the aqueous extract of *D. integrifolia* (100 mg/kg and 200 mg/kg) significantly (*p* < 0.01) raised the discrimination index to 0.30 ± 0.05 and to 0.5 ± 0.05, respectively. The treatment of mice with memantine also lead to a significant (*p* < 0.001) increased in the discrimination index to a value of 0.65 ± 0.05 (Figure 3(b)).

### 3.3. Effect of *Dichrocephala Integrifolia* on Inflammatory Parameters of Alcohol-Treated Mice

**Figure 4:**
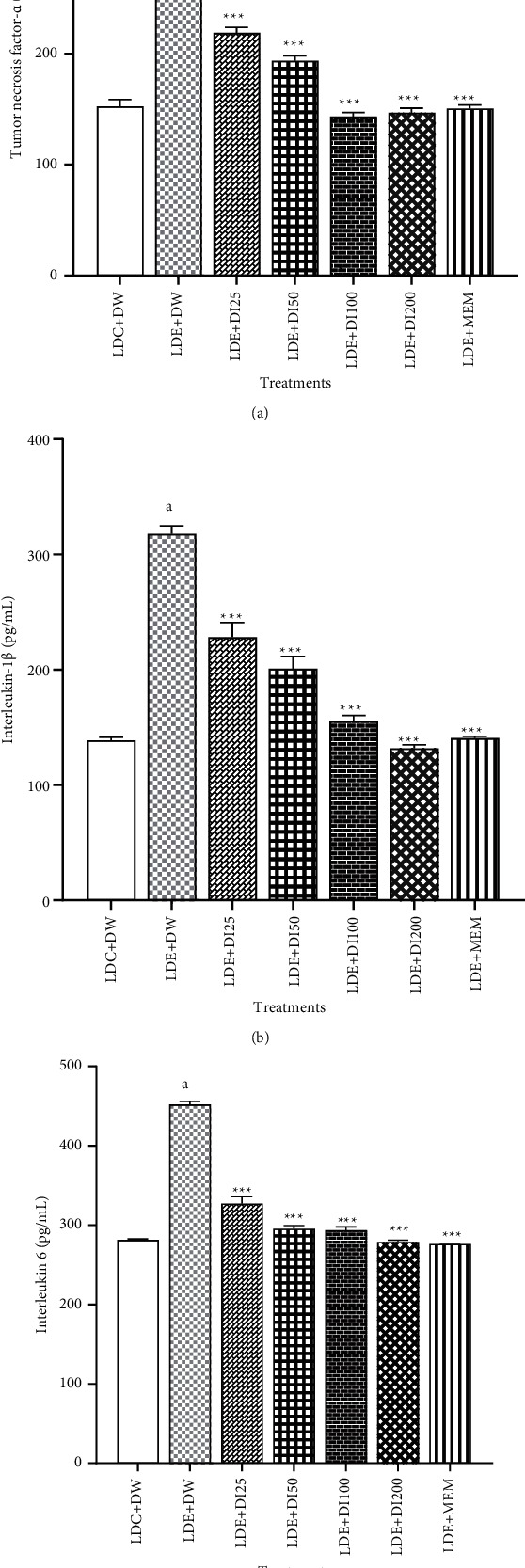
Effect of *Dichrocephala integrifolia* on inflammatory parameters of alcohol-treated mice. (a) Tumor necrosis-*α*. (b) Interleukin-1*β*. (c) Interleukin-6.

The results of the assay of inflammatory cytokines showed that the chronic treatment of mice with ethanol significantly increased the level of TNF-*α*, IL- 1*β*, and IL-6 (Figure 4).

The level of TNF-*α* was raised from 153 ± 6.04 pg/mL in the LDC + DW-treated group to 338 ± 5.92 pg/mL in the LDE + DW-treated group. This increase in TNF-*α* level was significantly lessened by *D. integrifolia*. The plant extract significantly (*p* < 0.001) reduced the level of TNF-*α* from 338 ± 5.92 pg/mL in the LDC + DW-treated group to 143 ± 3.57 pg/mL, and 147 ± 4.11 pg/mL at the doses of 100 and 200 mg/kg, respectively. Memantine (20 mg/kg) also decreased the TNF-*α* value to 151 ± 3.14 (*p* < 0.001) (Figure 4(a)).

Mice treated with alcohol in the LDE + DW group experienced a significant elevation in the level of IL-1*ß* in the brain. *D. integrifolia* significantly reversed this alcohol's effect in all doses of the treatment groups. The decreases in IL-1*β* were significant (*p* < 0.001) from 318. ± 6.88 pg/mL in the LDE + DW group to 156 ± 4.73 pg/mL; 132 ± 2.85 pg/mL and 141 ± 1.54 pg/mL in the plant at the doses, 100 and 200 mg/kg and memantine (200 mg/kg), respectively (Figure 4(b)).

*Dichrocephala integrifolia* significantly decreased the levels of IL-6 in the brains of mice that received chronic ethanol feeding with ethanol binges and this same reduction was noted in the group that received memantine as treatment. The level of IL-6 was significantly (*p* < 001) reduced from 452. ± 4 pg/mL in the LDE + DW-treated group to 294. ± 4.15 pg/mL, 279 ± 2.21 pg/mL and 276. ± 0.7 pg/mL in the plant at doses of 100 and 200 mg/ kg and memantine groups, respectively (Figure 4(c)).

Results are expressed as mean ± S.E.M (*n* = 8 animals) a*p* < 001 vs. LDE + DW; ^*∗∗∗*^*p* < 0.001 vs LDE + DW; DI25 : *Dichrocephala integrifolia* 25 mg/kg; DI 50: *Dichrocephala integrifolia* 50 mg/kg; DI 100: *Dichrocephala integrifolia* 100 mg/kg; DI 200: *Dichrocephala integrifolia* 200 mg/kg: DW: distilled water (10 ml/kg); LDC : Lieber-DeCarli control diet; LDE : Lieber-DeCarli ethanol diet; MEM : Memantine (20 mg/kg).

### 3.4. Effects of *Dichrocephala Integrifolia* on Some Biochemical Parameters of Oxidative Stress in Alcohol-Treated Mice

Table 2 shows the results of the effects of *D. integrifolia* on the brain levels of nitric oxide, malondialdehyde, reduced glutathione, and catalase activity. Twenty-eight (28) days of chronic ethanol feeding with ethanol binges resulted in an increase in the brain level of nitric oxide and malondialdehyde and a drop in the brain level of reduced glutathione and catalase activities in the LDE + DW-treated group when compared to the LDC + DW-treated group.

The brain nitric oxide level raised from 67.95 ± 6.42 mol/g of tissue in the LDC-treated group to 116.00 ± 3.26 mol/g in the LDE + DW-treated group (*p* < 0.001). *D. integrifolia* at the dose of 200 mg/kg as well as memantine significantly (*p* < 0.001) reduced this amount to 63.14 ± 2.57 mol/g and 62.15 ± 2.65 mol/g, respectively (Table 2).

The level of malondialdehyde was significantly (*p* < 0.001) lowered from 246.34 ± 6.86 *μ*mol/g in the LDE + DW to 145.40 ± 9.30 *μ*mol/g and 149.12 ± 9.63 *μ*mol/g at the dose of 100 mg/kg of *D. integrifolia* and memantine, respectively (Table 2).

The concentration of brain reduced glutathione lowered by chronic ethanol administration in the LDE + DW control group (12.61 ± 1.07 *μ*mol/g) was significantly (*p* < 0.05) raised by an administration of *D. integrifolia* at the dose of 200 mg/kg to a value of 50.55 ± 14.21 *μ*mol/g (*p* < 0.01) (Table 2).

The plant extract at the dose of 200 mg/kg significantly (*p* < 0.001) increased the brain level of catalase to 14.18 ± 1.84 mmol/g which was reduced in the LDE + DW-treated group to 1.33 ± 0.30 mmol/g. Memantine also significantly (*p* < 0.01) raised catalase activity to a value of 11.61 ± 1.75 mmol/g (Table 2).

## 4. Discussion

The present study was designed with the purpose, to assess the effects of an aqueous extract from the leaves of *D. integrifolia* on a mouse model of chronic ethanol feeding plus ethanol binges-induced oxidative stress, neuroinflammation, and memory dysfunctions in Swiss mice. The Lieber-DeCarli ethanol diet, which is a valid model to induce ethanol-use disorders [49] was used. Chronic ethanol exposures are among the leading causes of learning and memory disabilities, spatial working memory impairment as well as cognitive deficits [50, 51].

The results we obtained showed that 28 days of chronic ethanol feeding and ethanol binges twice a week have led to a profound alteration of memory in mice. Our results are in line with literature that has shown that excessive alcohol intake affects both short-term and long-term memory [52–54]. In fact, ethanol due to its ability to disrupt ionic contents of neurons dampens memory by slowing down communication between nerve cells of the hippocampus [3]. Murine models and behavioral tests have become increasingly, key tools in the investigation of ethanol-misuses-induced cognitive dysfunctions [10, 24, 50]. The *Y-*maze and the novel object recognition tasks are among the tools widely used in neuroscience to understand the role of different brain parts, such as the hippocampus, and the prefrontal cortex, in cognitive functions [38, 55–57].

In this study, fourteen days of chronic ethanol administration plus binges led to a reduction of the spontaneous alternation percentage in the negative control group. These findings corroborate those of previous authors who postulated that chronic ethanol administration impairs spontaneous alternation in the *Y* maze [58], and that the prefrontal cortex [50] and the hippocampus [13] are the brain area very sensitive to chronic ethanol consumption and ethanol binges. *D. integrifolia* significantly induced an increase in the percentage of spontaneous alternations across all treatment groups. These results are in agreement with those of Kouémou et al., in 2017 who reported that *D. integrifolia* increases the percentage of spontaneous alternations of mice in *Y*-maze. In nature, animals exhibit different behaviors in order to search for food resources and to run away from dangers [59]. Thus, there is a need for good learning and memory skills. In the present-day study, the novel object recognition task was used to assess the recognition memory of mice subjected to chronic ethanol administration, after 26 days of chronic ethanol administration. The results obtained validated the memory impairment already detected in the *Y* Maze. In fact, we noticed a rise in the exploration time of the familiar object over the novel object and a decrease in the discrimination index of mice subjected to chronic ethanol consumption. These results are in line with previous reports that revealed that binge-ethanol treatment induced spatial and recognition memory impairment even one-week later after the binge episode [60, 61].

The actions of ethanol on the learning and memory capacities of mice were significantly reversed by both the plant extract and memantine. These results once more showed the beneficial effects that have *D. integrifolia* extract to protect against memory impairment, as previously reported by Kouémou and coworkers in 2017 [32]. This effect of the plant can be partially explained by the fact that it is rich in chemical constituents such as flavonoids well acknowledged for their memory-enhancing effects [62]. The fact that the chronic administration of Lieber-DeCarli ethanol diet plus ethanol binges impaired spatial short-term memory and recognition memory in this work is in line with previous findings [63]. Da Silva and coauthors in 2018, also reported that alcohol causes miscommunication between neurons that generates toxicity. Neurotoxicity then decreases astrocyte and microglia density in the hippocampus thus, leading to memory deficits [3]. The results of these behavioral tests are converging proof that *D. integrifolia* has protective effects against chronic ethanol consumption and ethanol binges-induced memory impairment in mice.

Amongst the leading postulates behind the possible mechanisms by which alcohol leads to memory impairment are the oxidative stress and the neuroinflammation hypothesis of ethanol toxicity [50, 64]. In fact, chronic ethanol exposure results in an elevation of free radicals formation and reduction of anti-oxidant enzymes which in turn lead to memory impairment [25]. The results of the assay of oxidative stress parameters in this study are in line with those of Ngueguim et al. that showed an increase, in oxidative stress parameters of ethanol-treated mice and a protective effect of *D. integrifolia* against ethanol-induced oxidative stress [33]. Even though the investigations of these authors were at the level of the liver, a previous study has also shown that *D. integrifolia* protects brain against D-galactose-induced oxidative damage through its anti-oxidant properties [37]. The anti-oxidant properties of this plant are due to the presence of some chemical compounds such as flavonoids, tannins, and anthraquinones [43]. Growing lines of evidence have linked brain oxidative stress and neuroinflammation to ethanol toxicity [65]. In fact, ethanol metabolism leads to the production of reactive oxygen species and nitric oxide, these two end products are responsible for the induction of neuroinflammation via the regulation of the nuclear factor E2 related factor 2-heme oxygenase [65]. It is also well established that chronic alcohol abuse increases brain levels of cytokines either directly or indirectly by the pathway of peripheral inflammation [8, 50]. In fact, chronic ethanol elicits dynamic changes in TNF-*α*, in the cerebellum. TNF-*α* contributes to oxidative stress at the sites of inflammation [64]. The treatment of mice with alcohol significantly increased the brain level of TNF-*α*. This increase in TNF-*α* was significantly counteracted by *D. integrifolia* as well as the memantine-treated group. These results are in line with the literature that has demonstrated that chronic alcohol consumption increases the brain level of TNF-*α* [8]. The fact that *D. integrifolia* inhibited this elevation of brain level of TNF-*α*, suggests that *D. integrifolia* has an anti-inflammatory property. *D. integrifolia* could therefore act directly by reducing the brain cytokines level as related in this study; or indirectly by the reduction of inflammatory cytokines at the level of the liver as previously described [33]. Alcohol-induced neuroinflammation is also mediated by proinflammatory cytokine IL-1*β* in the brain. IL-1*β* exerts a number of diverse actions in the brain which contributes to neurodegeneration [67]. Our findings showed that 28 days of chronic ethanol feeding plus binges twice a week led to an increase in the level of IL-1*β* in the mice's brains. These results are similar to those obtained by Lowe and collaborators in 2020. *D. integrifolia* aqueous extract as well as memantine significantly reversed this alcohol's effect suggesting once more an anti-inflammatory effect. Memantine is an N-methyl-D-Aspartate receptor antagonist that has been used for years in the treatment of human alcoholism [68]. One of the key mechanisms by which memantine exerts its effects is through the blockage of the overstimulation of glutamate receptors and the subsequent neuroinflammation and neurodegeneration [15]. It is also well established that the overstimulation of N-methyl-D-aspartate receptor glutamate receptor is a mechanism by which alcohol leads to neuroinflammation and memory impairment [60, 69, 70]. IL-6 is also among the key pro-inflammatory cytokines involved in chronic ethanol exposure [71]. It is also a multifunctional cytokine that plays a critical role in the pathogenesis of inflammatory disorders and in the physiological homeostasis of neurons [72]. In the current study, chronic ethanol feeding plus ethanol binges led to an increase in the levels of IL-6. *D. integrifolia* and memantine significantly decreased the brain levels of IL-6 confirming the efficacy of these treatments against alcohol-induced neuroinflammation.

In the field of alcoholic research, studies have reported a positive correlation between brain oxidative stress, ethanol-induced neuroinflammation [73], and memory impairment [69]. The overall results obtained during our evaluations corroborate these previous findings. The fact that the animal model used in this study reproduces some of the behavioral patterns of chronic ethanol consumption and that our plant extract at different doses antagonizes it, is good news for the treatment of human alcoholism. Thus, the main finding of this research is that *D integrifolia* counteracted spatial short-term memory and recognition memory induced by chronic alcohol intake. The overall mechanism by which *D. integrifolia* protects against ethanol toxicity is through its anti-oxidant and anti-inflammatory properties. The results on this.

## 5. Conclusion

At the end of this investigation, we can undoubtedly say that the results of this study point to new findings about the potential benefits of *D. integrifolia* as a possible new therapeutic agent for the management of human alcoholism consequences. Nevertheless, for a better valorization of this plant in the treatment of ethanol addiction, it will be worthy to test its effects on other models of alcoholism like ethanol withdrawal syndrome and fetal alcohol spectrum disorders.

## Figures and Tables

**Table 1:** Animal grouping.

Groups	Treatments
LDC + DW	Lieber-DeCarli control diet + 10 ml/kg of distilled water
LDE + DW	Lieber-DeCarli ethanol diet + 10 ml/kg of distilled water
LDE + DI25	Lieber-DeCarli ethanol diet + 25 mg/kg of *D. integrifolia*
LDE + DI50	Lieber-DeCarli ethanol diet + 50 mg/kg of *D. integrifolia*
LDE + DI100	Lieber-DeCarli ethanol diet + 100 mg/kg of *D. integrifolia*
LDE + DI200	Lieber-DeCarli ethanol diet + 200 mg/kg of *D. integrifolia*
LDE + MEM	Lieber-DeCarli ethanol diet + memantine (20 mg/kg)

DI: Dichrocephala integrifolia; DI25: Dichrocephala integrifolia 25 mg/kg, DI50: Dichrocephala integrifolia 50 mg/kg, DI100: Dichrocephala integrifolia 100 mg/kg, DI200: Dichrocephala integrifolia 200 mg/kg, DW: distilled water (10 mL/kg); LDC: Lieber-DeCarli control; LDE: Lieber-DeCarli ethanol; MEM: Memantine.

**Table 2:** Effect of *Dichrocephala integrifolia* on the brain level of NO, MDA GSH, and CAT levels.

Treatments (mg/kg)	Nitrite oxide level (mol/g tissue)	Malondialdehyde (*μ*mol/g tissue)	Glutathione (*μ*mol/g tissue)	Catalase (mmol/g tissue)
LDC + DW	67.95 ± 6.42	173.80 ± 12.72	90.44 ± 6.68	16.84 ± 4.58
LDE + DW	116.00 ± 3.26^*∗∗∗*^	246.34 ± 6.86^*∗∗∗*^	12.61 ± 1.07^*∗∗*^	1.33 ± 0.30^*∗∗∗*^
LDE + DI25	123.80 ± 4.24	215.43 ± 11.76	32.66 ± 21.29	3.40 ± 1.56
LDE + DI50	102.00 ± 6.84	157.43 ± 9.63^c^	26.14 ± 11.53	9.84 ± 1.55^a^
LDE + DI100	92.77 ± 5.56^b^	145.40 ± 9.30^c^	12.01 ± 1.78	10.37 ± 2.55^a^
LDE + 200	63.14 ± 2.57^c^	187.74 ± 8.88^b^	50.55 ± 14.21^a^	14.18 ± 1.84^c^
LDE + MEM	62.15 ± 2.65^c^	149.12 ± 9.63^c^	85.29 ± 8.11^b^	11.61 ± 1.75^b^

Results are expressed as mean ± S.E.M (*n* = 8 animals). ^*∗∗*^*p* < 0.001, ^*∗∗∗*^*p* < 0.001 vs. LDC + DW and ^a^*p* < 0.05, ^b^*p* < 0.01, ^c^*p* < 0.001 vs. LDE + DW; DI25: *Dichrocephala integrifolia* 25 mg/kg; DI 50: *Dichrocephala integrifolia* 50 mg/kg; DI 100: *Dichrocephala integrifolia* 100 mg/kg; DI 200: *Dichrocephala integrifolia* 200 mg/kg: DW: distilled water (10 ml/kg); LDC: Lieber-DeCarli control diet; LDE: Lieber-DeCarli ethanol Diet; MEM: Memantine (20 mg/kg).

## Data Availability

The data recorded and analyzed during this study are available from the corresponding author upon reasonable request.
